# A salp bloom (Tunicata, Thaliacea) along the Apulian coast and in the Otranto Channel between March-May 2013

**DOI:** 10.12688/f1000research.2-181.v1

**Published:** 2013-09-10

**Authors:** Ferdinando Boero, Genuario Belmonte, Roberta Bracale, Simonetta Fraschetti, Stefano Piraino, Serena Zampardi

**Affiliations:** 1 Universita' del Salento, DiSTeBA, 73100 Lecce, Italy; 2 National Research Council, Institute of Marine Sciences, 16149 Genoa, Italy

## Abstract

Between March-May 2013 a massive *Salpa maxima* bloom was recorded by a citizen science study along the Ionian and Adriatic coast of the Salento peninsula (Italy). Citizen records were substantiated with field inspections along the coast and during an oceanographic campaign in the Otranto Channel.

Salps clogged nets, impairing fishing activities along the coast. Swimmers were scared by the gelatinous appearance of the salps, and thought they were jellyfish. At the end of the bloom the dead bodies of the colonies, that were up to 6-7 m long, were accumulated along the coast and stirred by the waves, forming foams along dozens of kilometers of coast. The bloom also occurred at the Tremiti Islands, north of the Gargano Peninsula. The possible impacts of such events on the  functioning of pelagic systems are discussed.

## Observation

The phylum Chordata is divided into three subphyla, the invertebrate Tunicata and Cephalochordata, and the vertebrate Vertebrata. The tunicates comprise the popular class Ascidiacea, the sea squirts, the inconspicuous Appendicularia and Sorberacea, and the conspicuous Thaliacea. Thaliaceans are mostly planktonic and are part of gelatinous macrozooplankton (Bone, 1998) ^1^. They are usually open water animals and are rarely seen from the shore. They are filter feeders and their colonies can become rather big, reaching several metres in length. Salps are colonial thaliacea thriving in all oceans, their chains of zooids can be very conspicuous.

During the spring of 2013, a citizen science study ( http://meteomeduse.focus.it/), aimed at documenting the presence of gelatinous plankton in Italian waters (Boero 2013a) ^2^, recorded about sixty sightings of *Salpa maxima* Forskål, 1775. The first, scattered records of salps arrived from the Apulian coasts in March 2013. Records increased in April and reached a peak in May. In April-May 2013, some of the authors had several chances to document the bloom, from both the coast (FB, RB, SF, SP), and from an oceanographic vessel (GB) during a cruise for the project CoCoNet ( http://www.coconet-fp7.eu/), between 8–21 May 2013. In mid-May 2013 the colonies experienced massive degeneration and, in the following days, became stirred and stranded along parts of the Apulian coast where they formed foam belts, each several kilometers long.

**Figure 1.**
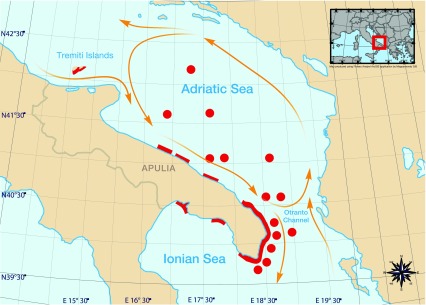
The peak of salp presence (early May 2013) along the Apulian coast. Coastal presences from both citizen science records and authors’ own observations were aggregated with the software Fishery Analyst, offshore records derive from onship observations during the CoCoNet Cruise. Yellow lines: trajectories of the main currents.

Figure 1 shows the distribution of the highest number of recorded salp chains of the citizen science study and of original observations by the authors in the first half of May, 2013. Figure 1 also shows the records of salp chains at stations of the CoCoNet cruise in the Otranto Channel. Dozens of colonies were visible from a single point of observation and, while moving, the same pattern persisted in a rather homogeneous fashion, leading to the perception of a single, large bloom, probably involving several million individuals. The bloom was more apparent from the coast, since the colonies were concentrated by the wind and currents, whereas in the open sea they were more scattered.

**Figure 2.**
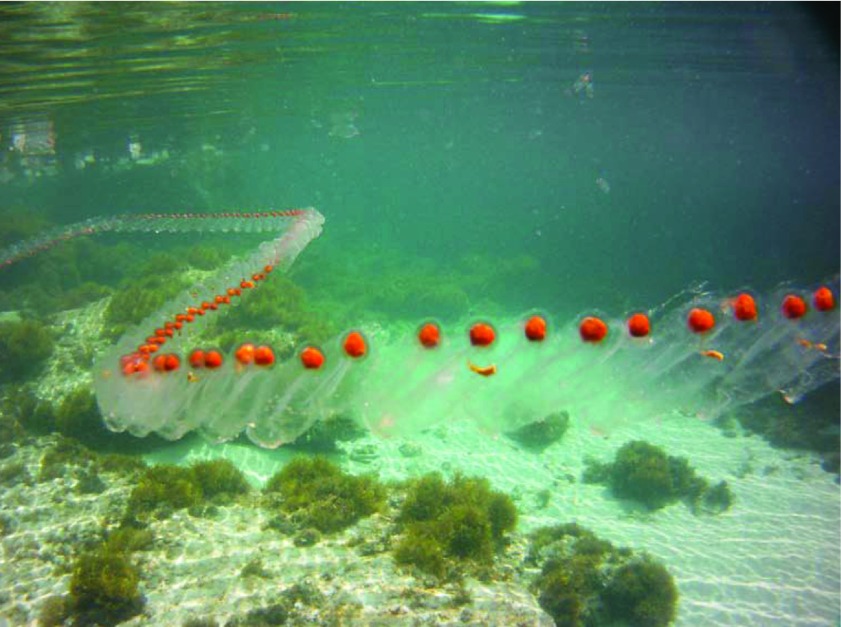
A chain of *Salpa maxima* in the waters of Salento. Picture by R. Bracale.

**Figure 3.**
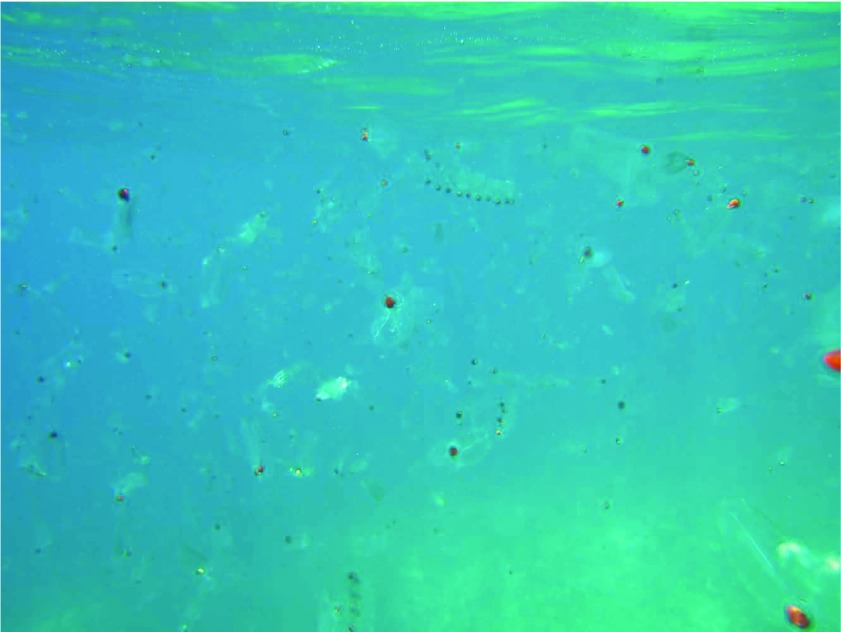
The fragments of salp chains, at the end of the bloom. Picture by S. Fraschetti.

The colonies were impressively long ( Figure 2) reaching up to 6–7 m in length and were seen by hundreds of people, including fishermen, who alerted the local authorities. At the end of the bloom, in mid-May 2013, the dissolving colonies were seen along the whole coast of the Salento pensinsula (the southernmost part of Apulia) ( Figure 3).

Merging the citizen science records with our direct observations from the coast and onboard the CoCoNet cruise, it is clear that the bloom was not a localized event, and that it characterized the plankton of a vast body of water for a prolonged period of time. During the same period of the reported bloom, citizens delivered just one salp record from the northern Adriatic and four from the Ligurian Sea, even though citizens sent many records of other gelatinous plankters, mostly jellyfish. The small number of salp records, and the massive presence of other gelatinous plankters along the whole coast of Italy (more than 5000 records from March to August 2013), are rather strong indications that salps were rare elsewhere beyond the Apulian coast. Since salp colonies ranged between 2 and 6–7 m in length, it is rather unlikely that organisms of that size might have passed unnoticed while much less conspicuous gelatinous plankters had been recorded.

## The public reaction

Many people thought that salps were jellyfish. Ocean literacy is rather limited, and very few people know what a salp is. People took pictures of them and reports started to appear in the local media, from local televisions to local newspapers and web sites. Eventually, the news reached the national press as well, with mentions in magazines such as *Internazionale* and *Venerdì di Repubblica*. A picture of a large colony, taken during a field inspection, became rather popular and was published several times ( Figure 2). Fishermen started to complain, because their nets were clogged and they could not operate. They documented the events with pictures, to support their requests of compensation.

The foam formed at the end of the bloom was at first attributed to a massive malfunction of sewage treatment plants, inexplicably occurring simultaneously in several plants that are independent of each other. The local media covered this event with some alarm. F. Boero made several interventions on local TVs and newspapers, explaining the reason of the appearance of the foam in such a wide area.

## Why a salp bloom can be important

Salps are gelatinous filter feeders; they are usually rather rare in Apulian waters, especially in coastal waters, and decades can pass without any salp observations. Records of salps in the scientific literature are rather scant, suggesting a similar pattern of occurrence also elsewhere. On June 5th 1998, F. Boero saw a bloom similar to the present one while crossing the Adriatic sea from Dubrovnick to Bari. During the six-hour journey, salps were constantly present in high densities, forming chains whose estimated length was 5–6 m. No report of that event was ever published. On that occasion, however, the colonies did not reach the coast, and remained off shore, with only relatively low quantities being washed ashore (the majority of them had ended their life cycle while sinking to the bottom).

The attention the 2013 salp bloom received in the media suggests that it is not likely that events of this magnitude occurred often in the past, since they would have been covered by the media as well. The surprise expressed by lay people and fishermen, indicates that, even if people are used to jellyfish, they are not used to salps, and the reason is simple: salps are rarely encountered in Italian waters. Such conspicuous animals do not pass unnoticed, and if they are around they are seen and recorded.

Salps have extremely high clearing rates and can feed on all sizes of phytoplankton, from viruses to protists, competing with crustacean filter feeders (Bone, 1998) ^1^. The presence of a massive bloom of salps likely depletes phytoplankton production, impairing the phytoplankton-crustacea-fish larvae and juveniles pathway (see Boero *et al.* 2008) ^3^. The match of fish recruitment with a salp proliferation, could lead to a decrease in fish reproductive success due to food depletion. If this phenomemon was not reported, it could result in fishery scientists who, after a few months, might record anomalies in the age classes of some fish species while being unable to link them to any particular cause.

Since salps tend to disgregate and precipitate as marine snow, they tend to fuel the detritus food chain on the sea bottom. Salp blooms, thus, might redirect the functioning of marine ecosystems in a sudden and dramatic way, leaving little evidence of the cause of changes that, indeed, might become apparent long after the end of the bloom.

As remarked by Boero (2013 b) ^4^, the availability of records of unusual events such as this salp proliferation, might help us to understand anomalies in the population structure of some species or even anomalies in the functioning of whole ecosystems.

## Predictability of salp blooms

Deibel and Paffenhöfer (2009) ^5^, whilst analysing a long time series of plankton records, identified a correlation between specific oceanographic conditions and the occurrence of thaliacean blooms, especially in correspondence with eddies, since these currents presumably concentrate salps. The area where the presently recorded salp bloom occurred is, indeed, characterized by a large gyre ( Figure 1). The current that flows along the Italian coast, and outflows from the Adriatic into the Ionian Sea is compensated by an inflow current that brings Ionian waters into the Adriatic sea on the East coast of the Adriatic (i.e. Greece, Albania, Montenegro, Bosnia and Croatia). These two opposite currents often generate a gyre in the Southern Adriatic, connecting the Eastern Adriatic coast to the Western one, immediately below the Gargano Peninsula, whereas, in correspondence of the Otranto Channel, a current connects the Italian Adriatic coast to the Albanian coast. The salp bloom, however, was also recorded at the Tremiti Islands, north of the Gargano Peninsula, and along the Ionian coast of the Salento Peninsula (e.g. Porto Cesareo) so outside the southern Adriatic gyre. Unfortunately, this area of the Adriatic is not systematically monitored for gelatinous plankton blooms, so the regularities identified by Deibel and Paffenhöfer (2009) ^5^, which occured along the Atlantic coast of the USA, cannot be searched for in Adriatic historical records. Licandro *et al.* (2006) ^6^, however, identified some correlations between hydrographic conditions and thaliacean presence along the Mediterranean coast of France, thus showing that the presence of thaliacean blooms is far from rare, under a given set of conditions, in spite of the scantiness of records. Both Licandro *et al.* (2006) ^6^ and Deibel and Paffenhöfer (2009) ^5^ ascribed the occurrence of thaliacean blooms to the occurrence of a given set of abiotic conditions, disregarding biotic interactions.

## Gelatinous plankton, ecosystem functioning and natural history

Thaliacean specialists, as Bone (1998) ^1^ summarized, concur that the ecological role of pelagic tunicates is much underestimated in plankton studies and that their impact on ecosystem functioning is much larger than commonly perceived.

The functioning of planktonic ecosystems is one of the most important ecological processes on the planet both in terms of oxygen production and as a carbon sink. The whole functioning of what is commonly called “nekton” (e.g. marine vertebrates and large squids), furthermore, depends on phytoplankton primary production and on the efficiency of energy transfer by grazers such as crustaceans or, as in this case, thaliaceans.

The presences of gelatinous plankton species, either carnivorous (jellyfish and ctenophores) or herbivorous (thaliaceans), commonly occurs in pulses that are almost invariably perceived as episodic and irregular events (Boero *et al.* 2008) ^3^ and so considered of negligible importance. This perception is simply wrong and, as Ricklefs (2012) ^7^ convincingly showed, the understanding of the functioning of ecosystems would gain much from the practicing of natural history. Regularities, such as the massive presence of crustacean plankton after phytoplankton blooms, do greatly attract ecologists, whereas irregularities such as gelatinous plankton blooms that, usually, are of short duration and might even be overlooked by standard monitorings, tend to be disregarded. If only regularities were responsible of the functioning of ecological systems, however, there would be no change. Irregularities are the motor of change and cannot be considered as negligible freaks, they are, instead, the key for the understanding of change, i.e. of the history of natural systems.
