# Supplementary material for: A salp bloom (Tunicata, Thaliacea) along the Apulian coast and in the Otranto Channel between March-May 2013
Source: F1000Res. 2013 Sep 10;2:181. [Version 1] doi: 10.12688/f1000research.2-181.v1 (PMC4647696; doi:10.12688/f1000research.2-181.v1)
Supplement: Peer review history

# Peer review: Referee response for version 1

**Published:** 2013-09-27

**Authors:** Priscilla Licandro

**Affiliations:** 1 Sir Alister Hardy Foundation for Ocean Science (SAHFOS), Plymouth, UK

This is an interesting and well written article, which reports a large bloom of gelatinous plankton (i.e. salps) observed in Spring 2013 along the Apulian coast (southern Adriatic). Such an exceptional event, which occurred for more than 2 months over a very large region, likely had a significant impact on the functioning of the marine ecosystem of the southern Adriatic, in particular on the proportion of sinking carbon as opposed to the carbon transferred across the pelagic food web.  Records of salp blooms are quite scant in the literature. For various reasons gelatinous zooplankton have been often overlooked so that, as rightly mentioned by the authors, blooms such as this have likely occurred but not been reported in the past. 

 Citizen science records, used in this study, have the advantage of providing real time information over a large spatial scale, a scale which is not covered by any monitoring program ongoing around the southern Adriatic or in other regions of the Mediterranean. Even though those records are not always rigorous measurements, and should be considered with care, I recommend that the authors provide more information on the range of Salpa maxima abundance observed in Spring 2013. Ranges of abundance (i.e. <10, 10-100, 100-500, 500-1000, >1000) available from the online portal http://meteomeduse.focus.it/ and similar - if not more precise information from the CoCoNet cruise - should be indicated, as they might enable researchers to assess whether S. maxima swarms were evenly distributed inshore and offshore, or whether they were instead concentrated in a particular area. 

I suggest some amendments in the text (see below for details), to clarify that salps are commonly found in Italian waters (see for instance the time series off Chiavari (Ligurian Sea) in Licandro & Ibanez, 2000 and off Naples (Tyrrhenian Sea) in Mazzocchi et al., 2011), although salp blooms are not regular events. In particular Salpa maxima, as well as other species of salps, had been previously found in the southern Adriatic (see ‘Checklist of the Flora and Fauna in Italian Seas-II’, 2010, Biologia Marina Mediterranea, Vol 17 (suppl.1)). The authors might want to mention that records of salps available in other regions of the southern Adriatic (e.g. Lokrum, the monitoring station off Dubrovnik, where gelatinous plankton has been regularly sampled every month since the mid-1990s) could possibly provide background information, to clarify the recurrence of salp blooms in the southern Adriatic and the environmental conditions associated with those events.  

Detailed comments:

1. Fig. 1 - I suggest indicating the average range of Salpa maxima abundance using, if necessary, different colours for different classes of abundance.
2. First paragraph, page 4 - I suggest the following modification: "The small number of salp records, and the massive presence of other gelatinous plankters along the whole coast of Italy (...) suggest that salps were rare elsewhere beyond the Apulian coast..."
3. Fourth paragraph, page 4 - I suggest the following modification: “Salps are gelatinous filter-feeders; they tend to be less abundant in Apulian waters..”
4. Fifth paragraph, page 4 - I suggest the following modification: “ ..blooms of salps are not regularly  encountered in Italian waters..”  

I have read this submission. I believe that I have an appropriate level of expertise to confirm that it is of an acceptable scientific standard.

# Peer review: Referee response for version 1

**Published:** 2013-09-26

**Authors:** Claudia Mills

**Affiliations:** 1 Department of Biology, University of Washington, Seattle, WA, USA

Salp blooms are medium-scale oceanographic events that happen normally throughout the world oceans. We can only appreciate the regularity, extent, and significance of these events if there is a record of their occurrence. This paper localizes an extensive salp bloom in time and space along the Apulian coast, and also mentions a single anecdotal observation of an unrecorded bloom in the same area in 1998, giving us perhaps an idea of the rarity of such an event.

I have read this submission. I believe that I have an appropriate level of expertise to confirm that it is of an acceptable scientific standard.
